# A Comparative Analysis of Lifetime Medical Conditions and Infectious Diseases by Sexual Identity, Attraction, and Concordance among Women: Results from a National U.S. Survey

**DOI:** 10.3390/ijerph16081399

**Published:** 2019-04-18

**Authors:** Kelly Horn, James A. Swartz

**Affiliations:** Jane Addams College of Social Work, University of Illinois at Chicago, Chicago, IL 60607, USA; jaswartz@uic.edu

**Keywords:** women’s health, sexual identity, chronic health conditions

## Abstract

There have been limited studies assessing the differences in chronic health conditions between sexual minority (those who identify as lesbian or bisexual) and sexual majority (heterosexual) women. Research has primarily focused on overall physical and mental health or behavioral issues and not on specific health conditions. The addition of sexual orientation and attraction questions to the National Survey on Drug Use and Health (NSDUH) now allows for research regarding health conditions using a national survey that identifies participant sexual orientation and attraction. This study sought to compare the prevalence/odds of having 10 medical conditions/infectious diseases among women, assessing for differences associated with sexual identity, sexual attraction, and the degree of concordance between sexual identity and attraction. Data from 67,648 adult female participants in the 2015–2017 NSDUH survey were analyzed using bivariate and multivariable logistic regression models to assess for differences in prevalence/odds of seven medical conditions. Multivariable models adjusted for demographics, substance abuse/dependence, and mental illness. We found significant differences by sexual identity, but not sexual attraction or concordance. Compared with heterosexually identified women, women who identified as bisexual had significantly higher odds of having three medical conditions and two infectious diseases than heterosexual or lesbian women. The findings generally support those based on studies using more limited geographical samples. There are a number of potential associated and underlying factors that contribute to bisexual women reporting overall poorer health than heterosexual or lesbian women. The factors discussed include stigma, delays in seeking care, lack of insurance and access, and sexual minority women receiving poorer health care generally.

## 1. Introduction

Research on health disparities among sexual minorities–those who identify as gay, lesbian, or bisexual–has focused on general physical well-being and the presence of mental health concerns such as depression and anxiety [1,2]. Other focal areas of health-related research among sexual minorities include behavioral health issues such as smoking, substance use, and binge drinking, as well as other health-related behaviors and social issues that affect health such as obesity, poor diet, inter-partner violence, stigma, and stress [3,4,5,6]. Until recently, there has been relatively little direct examination of the distal consequences of these behaviors and issues like the prevalence of specific chronic medical conditions (CMCs) such as heart disease, diabetes, asthma, or cancer in sexual minorities [7,8,9,10,11].

Gaining a complete understanding of the physical health of sexual minority men and women has been challenging due to the historical exclusion of questions on sexual identity from nationally representative health surveys. Consequently, earlier studies on sexual minority health were not nationally representative and restricted to using largely state-based data or data derived from broad-based surveys that nevertheless use state-based sampling such as the Behavioral Risk Factor Surveillance Study (BRFSS) [7,8,9,10,11,12,13]. The findings across these studies are difficult to compare, owing to the different sampling frames, instruments, and conditions assessed. However, in general, these studies most consistently found sexual minority participants, with results varying by gender, to be at risk for poorer health overall and at greater risk for poorer health given higher rates of behaviors such as smoking and substance use [3,8,14,15,16,17,18]. Less consistent were the findings for differing rates/odds of specific CMCs by sexual identity, which seem to vary the most from study to study [19].

The inclusion of measures related to sexual orientation, such as sexual identity and attraction, in surveys such as the National Health Interview Survey (NHIS) added in 2013 [20], and the National Survey on Drug Use and Health (NSDUH) added in 2015 [20] now permit broader and more detailed examinations of sexual minority health and specific CMC prevalence using nationally representative samples. For instance, a recent study using NHIS data found male and female sexual minority participants to have higher odds of multiple health risk factors (e.g., smoking, heavy drinking, psychological distress) and multiple CMCs than their heterosexual peers (but did not report on specific CMC prevalence) [10].

An understudied aspect of the association between sexual orientation and health is concordance between sexual identity and attraction, as health disparities could depend on the degree of concordance between the two [21]. Those whose sexual identity and attraction match are considered concordant, whereas those whose sexual identity and attraction do not match are discordant [21]. One might hypothesize that discordance is associated with increased stress and, consequently, individuals whose sexual identity is discordant with sexual attraction could be at higher risk for poor health. To the best of our knowledge, this hypothesis was only tested in a parallel study assessing the same data with exclusively men [22]. Using a nationally representative data set, this study sought to examine the relative health effects of sexual identity, attraction, and concordance comparing sexual minority and majority women on a set of CMCs as well as infectious diseases. The findings of a companion study of men using the same comparisons and parallel statistical analyses was published elsewhere [22]. This current study was done to examine the unique experiences of women.

### Objectives

The purpose of the current study was to determine the prevalence of chronic health conditions and infectious diseases among women, specifically assessing differences between sexual minority women and heterosexual women. This study examined the prevalence of health conditions and infectious diseases based on three distinct constructs: Sexual identity, sexual attraction, and concordance between sexual identity and attraction.

## 2. Method

This study was a secondary analysis of data collected for the 2015–2017 NSDUH. Details regarding the NSDUH methodology are available at the Substance Abuse and Mental Health Data Archive (https://www.datafiles.samhsa.gov/) [23].

### 2.1. Sample

As those under 18 are not asked questions about sexual identity or attraction, the sample was restricted to the 67,648 female participants aged 18 years or older. Sample size was reduced to 63,495 after removing 4153 (6.2%) participants with missing data on sexual identity and attraction. For the multivariable model with HIV/AIDS status as the dependent variable, sample size was further reduced to 63,346 owing to missing data for this variable (*N* = 149, 0.03%).

### 2.2. Measures

#### 2.2.1. Sexual Identity, Attraction, and Concordance

Sexual identity was evaluated using a three-category self-reported variable: Heterosexual, bisexual, or gay/lesbian. Sexual attraction was measured as a self-reported interval-level variable based on a single question that asked respondents to rate sexual attraction using a five-point scale that ranged from “only attracted to the opposite sex” (1) to “only attracted to the same sex” (5). A two-category variable was created to assess sexual concordance/discordance between sexual identity and attraction. Concordance was defined as identifying as heterosexual and reporting sexual attraction only to the opposite sex, identifying as gay or lesbian and reporting sexual attraction to only the same sex, or identifying as bisexual and reporting sexual attraction to either sex. Participants who did not meet these criteria were categorized as discordant, meaning there was some discrepancy between their identified sexual identity and sexual attraction.

#### 2.2.2. Demographics

The multivariable models controlled for the following demographics: Race/ethnicity (White, African-American/Black, Hispanic, and Asian/Pacific Islander/Multi-ethnic/Other); age (five categories from 18–25 years through 65 and older); education (four categories from less than high school through college graduate); marital status (married, widowed, separated/divorced, never married); poverty level (living in poverty, income up to twice the poverty level, and income greater than twice the poverty level); population density (living in a core-based statistical area (CBSA) with greater than a million people, living in a CBSA with fewer than a million, and not living in a CBSA). Population density is based on Core Based Statistical Areas (CBSAs), which are used by the U.S. Office of Management and Budget to determine population centers in the United States. Body mass index (BMI) was measured as an interval-level variable calculated from participant weight and height.

#### 2.2.3. Past-Year Mental Illness

Past-year mental illness was assessed as a four-category variable (none, mild, moderate, severe). This variable was based on predicted probabilities of having a mental illness and severity level of the mental illness given the participant’s age, level of functional disability, suicidal ideation, and major depressive episode based on NSDUH subsample validation studies that compared this measure with the results of clinical interviews using a semi-structured diagnostic instrument [24].

#### 2.2.4. Substance Abuse/Dependence

Alcohol and other drug abuse/dependence excluding nicotine were based on self-report and assessed with two binary (no/yes) variables reflecting past-year alcohol abuse/dependence and past-year abuse/dependence on drugs other than alcohol. Criteria for abuse/dependence were based on the Diagnostic and Statistical Manual of Mental Disorders, Fifth Edition (DSM-IV) diagnostic criteria [25]. Past-month nicotine dependence was also assessed as a binary variable based on participant responses to the Nicotine Dependence Syndrome Scale embedded in the NSDUH survey [26].

### 2.3. Dependent Variables

#### Medical Conditions and Infectious Diseases

NSDUH participants were asked if they were told by a doctor or other health care professional that they had any of the following eight medical conditions: Asthma, heart condition, hypertension, diabetes, chronic bronchitis or chronic obstructive pulmonary disease (COPD), cirrhosis, kidney disease, or any kind of cancer. Cirrhosis was dropped from the analysis due to the small number of participants who reported ever having that condition (*N* = 154). The survey also asked about the lifetime presence of any of the following infectious diseases: Hepatitis B or C; sexually transmitted infections (STIs) other than HIV/AIDS, such as syphilis, gonorrhea, chlamydia, or herpes; or HIV/AIDs. All medical conditions and infectious diseases included in the analysis were coded as binary (yes/no) variables.

### 2.4. Analyses

Analyses were conducted using Stata 14.2. (StataCorp LLC, College Station, TX, USA) [27]. Data were weighted for selection probability and standard errors were adjusted to reflect NSDUH design characteristics owing to clustering and stratification [24]. Bivariate analyses were first run to obtain the unadjusted prevalence rate for each medical condition or infectious disease by sexual identity. The bivariate prevalence rates for each medical condition were obtained by cross-tabulating the condition with the three-category variables used to represent sexual identity and obtaining the percentage positive for that condition within each sexual identity category. In the multivariable models, the adjusted odds ratios (AORs) of having each medical condition or infectious disease for sexual identity, attraction, and concordance were determined in separate binary logistic models. Interaction effects were also conducted to determine if there were any significant associations between sexual identity and concordance/discordance. Model covariates included: Demographics, mental illness, substance abuse/dependence, and nicotine dependence. All multivariable models also adjusted for HIV/AIDS, except in the model in which HIV/AIDS was the dependent variable.

## 3. Results

### 3.1. Demographics

Table 1 presents sample demographics by sexual identity. There were significant demographic differences by sexual identity for all demographic variables. Individuals between the ages of 18–34 were more likely to report being bisexual than any other age group (70%), whereas those who were 65 and older were much more likely to report being heterosexual (21.9%) than bisexual (2.4%) or lesbian (12.0%; F_(5.1, 253.9)_ = 131.8, *p* < 0.001). Bisexual women were more likely to report having some college (38.4%) than a high school degree (26.9%) or a college degree (21.8%) or some high school (13.0%; F_(4.8, 238.4)_ = 14.0, *p* < 0.001). Heterosexual participants were more like to report being married (51.5%), whereas those who identified as lesbian/gay (59.4%) or bisexual (58.4%) were more likely to have never been married (F_(4.23, 211.6)_ = 188.7, *p* < 0.001). Bisexual women also reported higher rates of poverty (26.4%) than lesbian/gay (18.9%) or heterosexual women (15.7%), with heterosexual women more likely to report incomes greater than twice the federal poverty level (63.4%; F_(3.3, 166.9)_ =40.2, *p* < 0.001). 

There was a strong and positive correlation between sexual identity and sexual attraction (*r* = 0.78, *p* < 0.01). Those who reported being attracted only to those of the opposite sex were also more likely to identify as heterosexual (91.6%), while those who identified as lesbian reported being only attracted to the same sex (56.1%) and mostly attracted to the same sex (32.3%; F_(6.3, 313.7)_ = 1125.7, *p* < 0.001). Women who identified as bisexual were the most likely to report concordance in their sexual identity and attraction (94.1%; F_(1.92, 96.0)_ =229.9, *p* < 0.001), followed by heterosexual (89.7%) and lesbian participants (55%). This result demonstrates that self-reported sexual identity may, in some instances, be in conflict with self-reported sexual attraction and may cause discordance.

### 3.2. Medical Conditions, Infectious Diseases, and Behavioral Health Issues

Table 2 shows the results for the bivariate comparisons of the unadjusted lifetime prevalence rates of the seven medical conditions and three infectious diseases by sexual identity. Several of the medical conditions showed significant differences attributable to sexual identity. Bisexual (18%) and gay/lesbian (16.5%) women were more likely than heterosexual (10.8%) women to report having asthma (F_(2.0, 99.4)_ = 36.4, *p* < 0.001). Conversely, lifetime prevalence of hypertension was reported more often by heterosexual women (22%) compared with lesbian (15.1%) and bisexual women (9.7%; F_(1.92, 96.1)_ = 84.9, *p* < 0.001).

Heterosexual women were also more likely to report having a heart condition (9.6%) than bisexual women (6.5%) or lesbian identified women (7.4%; F_(1.9, 98.8)_ =8.6, *p* < 0.01), and both lesbian women (10.1%) and heterosexual women (10.7%) were more likely than bisexual women (6.1%) to report having diabetes (F_(1.74, 87.1)_ = 15.5, *p* < 0.01).

There were significant associations between sexual identity and two of the three infectious diseases. Bisexual women (2%) reported higher rates of hepatitis B/C than gay/lesbian (1.5%) or heterosexual women (0.98%; F_(1.9, 99.0)_ =5.6, *p* < 0.01). Similarly, bisexual women were more likely to report having had an STI in the past 12 months (5.8%) compared to lesbian (2.2%) and heterosexual (2.2%) women (F_(1.9, 95.0)_ =46.7, *p* < 0.001). However, there was no significant difference in the prevalence of HIV/AIDS by sexual identity.

### 3.3. Multivariable Models of Medical Conditions and Infectious Diseases

Table 3 shows the AORs of having any medical condition or infectious disease for sexual identity, attraction, and concordance. The overall significance for each multivariable chronic condition model was assessed using the adjusted Wald F-test to account for any survey design effects [28]. There were significant associations between sexual identity and the odds of having three medical conditions and two of the three infectious diseases. Bisexual women (AOR = 1.5, *p* < 0.001) and lesbian women (AOR = 2.0, *p* < 0.05) had higher odds of ever having asthma than heterosexual women, while both bisexual (AOR = 1.8, *p* < 0.01) and lesbian/gay women (AOR = 3.0, *p* < 0.05) had higher odds of reporting chronic bronchitis/COPD relative to heterosexual women. Bisexual women were also more likely to report lifetime prevalence of any cancer (AOR = 1.9, *p* < 0.05) than heterosexual women. Among the infectious diseases examined, bisexual women had higher odds of reporting hepatitis B/C (AOR = 2.8, *p* < 0.05) and any STIs in the past 12 months (AOR = 2.1, *p* < 0.01) than heterosexual women.

To assess whether the effects of sexual identity varied by concordance/discordance status in influencing the prevalence of each chronic condition, the authors included terms representing the interaction between these two primary predictors in each multivariable model. The decision to retain the interaction as having additional influence was based on the significance level of the term, as well as comparison of standard model fit statistics, such as BIC and change in the log-likelihood ratio between the main effects only model and the model containing the interaction term. There was only a single significant difference for identity/attraction concordance: Women who were discordant regardless of sexual identity had higher odds of having STIs (AOR = 2.2, *p* < 0.001) than women whose sexual identity and attraction were concordant. There were no significant associations for any of the assessed conditions or infectious diseases and sexual attraction, although there was one significant interaction effect for sexual identity and concordance. Women who reported being bisexual and discordant (AOR = 0.2, *p* < 0.01) and gay and discordant (AOR = 0.2, *p* > 0.05) had higher odds of reporting STIs than those who had concordance between sexual identity and sexual attraction. 

## 4. Discussion

### 4.1. Main Findings

Similar to other studies that examined health disparities related to sexual identity [3,9,10,14,15,16,17,18,19], our analyses demonstrate differences in the prevalence and odds of a number of CMCs for sexual minority and majority women. Our results augment the findings of earlier studies by supporting their generalizability to a national sample. Most of the significant differences were among women identifying as bisexual in comparison to self-identified heterosexual women [29]. There were fewer significant differences among women identifying as lesbian/gay and no differences in health conditions based on sexual attraction. Although there was one significant difference between sexual minority women and sexual majority women based on concordance between sexual identity and sexual attraction, the overall pattern of results suggests that a person’s sexual identity has a greater bearing on health than attraction or the degree of concordance between identity and attraction.

There are likely a number of structural and psychosocial disparities that underlie the cross-study findings of poorer health among sexual minorities, particularly women, that are present in our results: Greater unaffordability of medical care due to health care insurance coverage, poorer quality of care, delays seeking medical care, and greater stress specifically due to identifying as a sexual minority. In this regard, our results could be viewed as supporting intersectionality theory whereby social and economic inequalities owing to race, gender, and sexual identity are related and have larger health effects than would be predicted if considered independently [30].

In our study as well as in previous research, bisexual women were more likely to be socioeconomically disadvantaged and living in impoverished conditions relative to other women [29]. Although the multivariable models in this analysis adjusted for poverty level, other poverty-related factors, such as poorer diet, could have increased the odds of having certain health conditions among bisexual participants and contributed to an inability to afford medical care. Blosnich et al. [31] found that bisexual and lesbian women had much lower odds of seeking medical care owing to cost compared to heterosexual women.

Sexual minority women reported receiving lower quality of medical care received even when they are able to afford care, leading to delays seeing a general practitioner or gynecologist [32]. Hesitation seeking medical care due to stigma and increased stress levels is particularly relevant for bisexual women who had the highest prevalence/odds for multiple health conditions. Research demonstrated that bisexual women report greater stigmatization and social exclusion within lesbian and gay communities as well as from heterosexual communities, resulting in less social support [33]. Greater social isolation could also account for bisexual women seeking more sexual partners in order to develop a sense of belonging, putting them at greater risk for STIs including infection with the human papilloma virus (HPV).

Stress among sexual minority women related to external prejudice, internalized homophobia, and environmental stress at work, school, or home have also been shown to affect health [34,35]. For instance, perceived prejudice related to sexual identity is associated with increased odds of experiencing physical health problems among LGB women even after adjusting for general stressful life events [34]. Additionally, the cumulative effects of persistently increased levels of stress in the lives of sexual minority women could contribute to deterioration in physical health over time. Our findings demonstrated that bisexual women, in particular, had increased odds of reporting any past-year mental illness, which could account for increased stress that may affect health and the development of chronic health conditions.

The findings of higher odds of CMCs and infectious diseases among sexual minority women stands in contrast to our parallel study of sexual minority men [22]. We found no significant associations between the same set of CMCs and infectious diseases and sexual identity, attraction, or concordance, with the exception of HIV/AIDS. The reasons for this gender-based discrepancy is unclear given that gay and bisexual men likely experience some of the same issues (e.g., stigma, medical care affordability) as sexual minority women warranting further study of these gender discrepancies.

### 4.2. Limitations

The NSDUH is cross-sectional and does not allow for assessment of health over the course of life. It is possible health disparities and the effects of sexual identity, attraction, and concordance change over time. Younger participants, especially those with a co-occurring mental illness or substance abuse issues, might not manifest adverse health consequences until later life. Additionally, self-report could generate under- or over-reporting of the CMCs or discrepancies in other measures like mental health, substance use, poverty level, or education. Individuals may have a condition of which they are unaware, particularly if it is asymptomatic or may report a condition that does not meet clinical diagnostic criteria. Furthermore, self-reporting can result in under- or over-reporting due to social desirability bias or recall bias [36]. Although this is a limitation, a review of the literature on the validity of self-reported health data found that self-reported health information is generally valid and reliable [37].

Another limitation is the absence of sexual behavior measures in the NSDUH. Although sexual identity and attraction are important, sexual behavior is another potentially important health-related dimension of sexuality [20]. For example, women who identify as bisexual may be attracted to both men and women but could be exclusively homosexual or heterosexual in their relationships. Questions on sexual behavior would be important additions to national surveys to gain a fuller picture of health related to different aspects of sexuality. Finally, only a limited number of CMCs were assessed with different results possible for excluded conditions.

### 4.3. Implications for Practice

Because both lesbian and bisexual women may not seek medical care to the extent that heterosexual women do, greater education should be given to physicians about sexual minorities and how best to care for them. Disclosing sexual identity to a physician can be seen as challenging as coming out to others for sexual minority patients [38]. Physicians should be mindful of creating a space that allows for disclosure of sexual identity and regularly asking individuals about their sexual preference in order to reduce stigma among sexual minorities and create a safe place for lesbian and bisexual women to share their health concerns. If sexual minority women feel more comfortable to seek care from a primary care physician or gynecologist, this may reduce the risk of developing health problems over time. Physicians who are working with bisexual women should be educated on the various implications and risks of having sexual partners of both sexes. Physicians who create a strong therapeutic relationship with sexual minority patients can enable those patients to discuss health issues more openly and disclose their sexual identity, leading to an increased comfort in seeking medical care as needed [38].

In addition, other professionals working with sexual minority women, such as social workers or counselors, should be aware of the potential increase of chronic health conditions and how that may impact treatment in therapeutic settings or settings that provide resources to sexual minority women. Providing resources for these women could have an impact for future health and providing a safe space for sexual minority women to share their concerns may help encourage women to seek medical help more regularly. If sexual minority women have greater odds of having chronic health conditions or infectious diseases, one potential way of reducing those odds is by creating spaces in which these women feel comfortable seeking medical or other care.

Future research should continue to explore the experiences of sexual minority women and health care. Studies that explore a larger number of CMCs, such as specific cancers, and which use longitudinal data to assess temporal changes would be especially valuable. Qualitative studies to assess the experiences of bisexual women seeking medical care to understand hindrances would allow development of strategies to foster higher engagement in medical care. Understanding health-related differences among sexual minority women related to poverty, racism, and stigma and how these intersect to produce poorer health is also critically important.

## 5. Conclusions

Using a nationally representative sample, we found significant differences in CMCs and infectious diseases for sexual minority in comparison to sexual majority women, especially those who identified as bisexual. Greater understanding and acceptance of bisexuality by both the heterosexual and gay communities could be beneficial in encouraging bisexual women to seek medical care or explore their sexuality in safe ways, which could help to prevent STIs or other CMCs from developing. Bisexual-specific organizations are on the rise [33] and future research could assess the extent to which these environments impact bisexual women’s health, tendency to seek medical care, and attitudes about sexuality.

## Figures and Tables

**Table 1:** Demographics and Self-reported Sexual Attraction by Sexual Identity.

Sexual Identity	Heterosexual		Gay or Lesbian		Bisexual		Totals		Sig
	(*N* = 62,038)		(*N* = 1321)		(*N* = 4289)		(*N* = 67,648)		
	%	[95% CI]	%	[95% CI]	%	[95% CI]	%	[95% CI]	
Race/Ethnicity									**
Non-Hispanic white	64.7	[63.9, 65.5]	63.8	[59.9, 67.8]	61.3	[59.4, 63.3]	64.6	[63.8, 65.3]	
Non-Hispanic black/African-American	12.4	[11.9, 13.0]	15.7	[13.0, 18.8]	14.1	[12.7, 15.7]	12.5	[12.0, 13.0]	
Hispanic	15.1	[14.5, 15.7]	14.3	[11.7, 17.4]	15.7	[14.2, 17.3]	15.1	[14.5, 15.7]	
Asian/Pacific Islander/Native American/multi-ethnic	7.9	[7.5, 8.2]	6.2	[4.6, 8.4]	8.9	[7.5, 10.5]	7.9	[7.5, 8.2]	
Age (in years)									***
18–25	12.4	[12.1, 12.8]	20.9	[18.5, 23.5]	41.5	[39.5, 43.6]	13.7	[13.3, 14.0]	
26–34	14.8	[14.5, 15.2]	20.6	[17.6, 24.1]	28.5	[26.6, 30.4]	15.5	[15.1, 15.8]	
35–49	24.6	[24.0, 25.1]	23.0	[20.1, 25.6]	19.8	[17.9, 21.9]	24.4	[23.9, 24.9]	
50–64	26.2	[25.6, 26.9]	23.5	[19.2, 28.4]	7.8	[5.9, 10.3]	25.5	[24.9, 26.1]	
65+	21.9	[21.2, 22.7]	12.0	[8.1, 17.4]	2.4	[1.6, 3.6]	21.0	[20.4, 21.7]	
Education (highest grade)									***
Less than high school	11.9	[11.3, 12.4]	10.4	[8.2, 13.3]	13.0	[11.8, 14.3]	11.9	[11.4, 12.4]	
High school graduate	23.5	[23.0, 24.0]	19.5	[16.5, 22.9]	26.9	[25.5, 28.3]	23.6	[23.1, 24.0]	
Some college/associate’s degree	32.8	[32.2, 33.4]	35.2	[31.1, 39.5]	38.4	[36.5, 40.3]	33.0	[32.5, 33.6]	
College graduate	31.9	[31.1, 32.7]	35.0	[30.3, 39.8]	21.8	[19.7, 24.0]	31.6	[30.8, 32.3]	
Marital Status									***
Married	51.5	[50.8, 52.3]	25.5	[20.9, 30.7]	24.8	[23.0, 26.6]	50.1	[49.3, 50.8]	
Widowed	8.9	[8.5, 9.3]	3.2	[1.7, 5.9]	1.1	[0.8, 1.5]	8.5	[8.1, 8.9]	
Divorced or Separated	15.9	[15.3, 16.4]	11.9	[9.9, 14.1]	15.7	[14.0, 17.7]	15.8	[15.3, 16.3]	
Never been married	23.8	[23.2, 24.3]	59.4	[54.3, 64.4]	58.4	[56.3, 60.5]	25.7	[25.1, 26.2]	
Poverty Level									***
Living in poverty	15.7	[15.2, 16.2]	18.9	[15.6, 22.6]	26.4	[24.8, 28.1]	16.2	[15.6, 16.7]	
Income up to twice the federal poverty level	21.0	[20.4, 21.4]	19.5	[16.3, 23.3]	24.3	[22.7, 26.0]	21.0	[20.5, 21.5]	
Income greater than twice the federal poverty level	63.4	[62.7, 64.1]	61.6	[56.8, 66.2]	49.3	[47.1, 51.5]	62.8	[62.1, 63.6]	
Population Density ^a^									**
CBSA > 1 million	53.5	[52.7, 54.2]	54.0	[49.4, 58.6]	55.9	[53.9, 58.0]	53.6	[52.9, 54.3]	
CBSA < 1 million	40.7	[39.9, 41.7]	42.1	[37.5, 47.0]	39.6	[37.9, 41.4]	40.7	[39.9, 41.4]	
Not in CBSA	5.9	[5.4, 6.4]	3.8	[2.5, 5.8]	4.5	[3.7, 5.5]	5.8	[5.3, 6.3]	
Sexual Attraction ^b^									***
Only attracted to opposite sex	91.6	[91.3, 91.8]	3.7	[2.1, 6.2]	5.6	[4.4, 7.3]	87.0	[86.7, 87.3]	
Mostly attracted to opposite sex	6.2	[6.0, 6.4]	3.5	[1.9, 6.3]	28.6	[26.7, 30.5]	7.0	[6.8, 7.2]	
Equally attracted to both sexes	1.6	[1.4, 1.7]	4.5	[3.0, 6.6]	59.4	[57.1, 61.7]	3.8	[3.6, 3.9]	
Mostly attaracted to same sex	0.1	[0.1, 0.2]	32.3	[28.9, 35.9]	6.1	[5.1, 7.2]	0.9	[0.8, 0.9]	
Only attracted to same sex	0.5	[0.5, 0.6]	56.1	[52.1, 60.0]	0.3	[0.1, 0.6]	1.4	[1.3, 1.6]	
Sexual Attraction (mean score)	1.1	[1.1, 1.1]	4.3	[4.2, 4.4]	2.7	[2.6, 2.7]	1.3	[1.1, 1.13]	***
Sexual Identity and Attraction									
Concordant	89.7	[89.4, 90.0]	55.0	[51.0, 58.9]	94.1	[92.4, 95.4]	89.3	[88.6, 89.9]	***
Discordant	10.3	[10.0, 10.6]	45.0	[48.1, 49.0]	5.9	[4.6, 7.6]	10.7	[10.1, 11.4]	

Note: All figures reflect weighted percentages and are based on 67,648 female NSDUH participants 18 years of age and older. Sample N’s at the top of each column are unweighted. All figures are percentages unless otherwise indicated. Design-based F-tests based on the weighted data and controlling for stratification and clustering were used to test statistical significance. **^a^** Population density is based on Core Based Statistical Areas (CBSA), which are used by the U.S. Office of Management and Budget to define population centers in the U.S. **^b^** Sexual attraction was self-reported using a scale from 1 to 5 with the lowest score indicating exclusive attraction to members of the opposite sex and higher scores indicating increasingly greater attraction to the same sex. 95% CI= 95% Confidence Interval. Sig=significance level. ** *p* < 0.01; *** *p* < 0.001.

**Table 2:** Bivariate Comparisons of the Lifetime Prevalence of Medical Conditions, Infectious Diseases, and Behavioral Health Issues by Sexual Identity.

Sexual Identity	Heterosexual		Gay or Lesbian		Bisexual		Totals		Sig
	(*N* = 62,038)		(*N* = 1321)		(*N* = 4289)		(*N* = 67,648)		
	%	[95% CI]	%	[95% CI]	%	[95% CI]	%	[95% CI]	
Medical Conditions									
Asthma	10.8	[10.5, 11.2]	16.5	[13.5, 20.0]	18.0	[16.1, 20.2]	11.2	[10.8, 11.6]	***
Heart condition	9.6	[9.2, 10.0]	7.4	[5.3, 10.2]	6.5	[5.4, 8.0]	9.4	[9.0, 9.8]	**
Hypertension	22.0	[21.5, 22.6]	15.1	[12.3, 18.4]	9.7	[8.4, 11.2]	21.4	[20.9, 22.6]	***
Diabetes	10.7	[10.3, 11.2]	10.1	[7.3, 13.8]	6.1	[5.1, 7.3]	10.5	[10.1, 11.0]	***
Chronic bronchitis or COPD	5.4	[5.1, 5.7]	6.9	[4.4, 10.6]	5.6	[4.7, 6.6]	5.5	[5.2, 5.8]	NS
Cirrhosis	0.2	[0.1, 0.3]	0.2	[0.003, 0.2]	0.3	[0.1, 0.9]	0.2	[0.2, 0.3]	NS
Kidney Disease	2.2	[2.0, 2.4]	1.3	[0.5, 3.2]	1.6	[1.1, 2.3]	2.1	[1.9, 2.3]	NS
Cancer (any kind)	7.3	[6.9, 7.6]	5.9	[3.7, 9.2]	3.9	[3.0, 5.2]	7.1	[6.8, 7.5]	**
Infectious Diseases									
Hepatitis B or C	1.0	[0.9, 1.1]	1.5	[0.6, 3.0]	2.0	[1.4, 2.8]	1.0	[0.9, 1.2]	**
Sexually transmitted infections ^a^	2.2	[2.0, 2.4]	2.2	[1.4, 3.4]	5.8	[5.0, 6.8]	2.3	[2.2, 2.5]	***
HIV/AIDS	0.1	[0.1, 0.1]	0.2	[0.1, 0.5]	0.1	[0.0, 0.4]	0.1	[0.1, 0.1]	NS
Behavioral Health Issues									
Body Mass Index	28.1	[28.0, 28.2]	29.1	[28.5, 29.6]	28.8	[28.4, 29.1]	28.7	[28.3, 29.0]	***
Past-year mental illness ^b^									***
Mild	10.4	[10.1, 10.7]	13.1	[10.6, 16.0]	17.1	[15.6, 18.8]	10.6	[10.4, 11.0]	
Moderate	5.6	[5.3, 5.8]	6.1	[4.8, 7.8]	14.0	[12.6, 15.5]	5.9	[5.7, 6.1]	
Serious	4.9	[4.6, 5.1]	12.6	[10.1, 15.6]	20.3	[18.6, 22.1]	5.6	[5.3, 5.9]	
Past-year substance abuse or dependence									
Nicotine	10.0	[9.6, 10.3]	16.2	[13.3, 19.6]	20.0	[18.4, 21.7]	10.5	[10.1, 10.8]	***
Alcohol	3.9	[3.7, 4.1]	7.7	[6.0, 9.9]	12.5	[11.2, 13.8]	4.3	[4.0, 4.7]	***
Other drugs	1.6	[1.5, 1.7]	4.3	[3.3, 5.7]	8.5	[7.4, 9.8]	1.9	[1.8, 2.0]	***

Note. All figures reflect weighted percentages and are based on 67,648 female NSDUH participants 18 years of age and older. Subgroup N’s at the top of each column are unweighted. All figures are percentages unless otherwise indicated. Design-based F-tests based on the weighted data and controlling for stratification and clustering were used to assess statistical significance. ^a^ Sexually transmitted infections includes gonorrhea, chlamydia, syphilis, or herpes. **^b^** Past-year mental illness category is based on thresholds derived from a logistic regression model of mental illness as predicted by age, functional disability, thoughts of suicide, and major depressive episode. 95% CI = 95% Confidence Interval. Sig = significance level. NS = non-significant; ** *p* < 0.01; *** *p* < 0.001.

**Table 3:** Bivariate Logistic Regression Results for Medical Conditions and Infectious Diseases.

Medical Condition/Infectious Disease	Asthma			Heart Condition			Hypertension			Diabetes			Chronic Bronchitis/COPD		
	AOR	[95% CI]		AOR	[95% CI]		AOR	[95% CI]		AOR	[95% CI]		AOR	[95% CI]	
Sexual Identity (Heterosexual = reference)															
Bisexual	1.5	[1.2, 2.0]	***	1.2	[0.8, 1.7]		1.0	[0.7, 1.4]		1.4	[1.0, 2.0]		1.8	[1.2, 2.7]	**
Gay	2.0	[1.1, 3.5]	*	0.7	[0.3, 1.7]		0.8	[0.4, 1.6]		1.8	[0.4, 4.6]		3.0	[1.2, 7.7]	*
Sexual attraction ^b^	0.9	[0.8, 1.0]		1.1	[0.9. 1.3]		1.0	[0.9, 1.1]		0.8	[0.7, 1.0]		1.0	[0.8, 1.1]	
Discordant sexual identity and attraction (Condordant = reference)	1.2	[1.0, 1.5]		1.1	[0.8, 1.4]		1.0	[0.7, 1.3]		1.3	[0.9, 1.8]		1.0	[0.7, 1.5]	
Sexual Identity and Concordance Interaction (concordant + bisexual discordant + gay discordant = reference)															
Bisexual and Discordant	0.5	[0.2, 1.0]	*	1.0	[0.4, 2.7]		0.6	[0.3, 1.5]		0.6	[0.2, 2.0]		1.3	[0.4, 4.5]	
Gay and Discordant	0.8	[0.5, 1.3]		1.1	[0.5, 2.3]		1.0	[0.4, 2.3]		1.0	[0.3, 2.6]		0.4	[0.1, 1.2]	
Race/ethnicity (White = reference)															
Non-Hispanic black/African-American	0.9	[0.8, 1.0]		0.6	[0.5, 0.7]	***	1.8	[1.6, 2.0]	***	1.6	[1.4, 1.8]	***	0.6	[0.4, 0.7]	***
Hispanic	0.8	[0.7, 0.9]	***	0.6	[0.4, 0.7]	***	0.8	[0.7, 0.9]	**	1.6	[1.4, 1.9]	***	0.5	[0.4, 0.7]	***
Asian/Pacific Islander/Native American/multi-ethnic	1.0	[0.8, 1.2]		0.7	[0.5, 0.9]	**	1.2	[1.1, 1.5]	*	1.8	[1.4, 2.3]	***	0.9	[0.7, 1.2]	
Age in years (18–25 = reference)															
26–34	0.8	[0.7, 0.9]	***	1.0	[0.8, 1.4]		2.3	[2.0, 2.7]	***	1.9	[1.6, 2.3]	***	1.4	[1.1, 1.8]	**
35–49	0.7	[0.6, 0.8]	***	1.8	[1.5, 2.0]	***	5.8	[5.1, 6.7]	***	3.9	[3.2, 4.7]	***	2.6	[2.1, 3.2]	***
50–64	0.7	[0.6, 0.7]	***	3.6	[3.0, 4.2]	***	16.7	[14.5, 19.3]	***	8.4	[6.9, 10.2]	***	6.7	[5.4, 8.3]	***
65+	0.7	[0.6, 0.8]	***	9.6	[8.2, 11.2]	***	37.6	[32.6, 43.5]	***	14.2	[11.6, 17.4]	***	12.9	[10.3, 16.3]	***
Education level (Less than high school = reference)															
High school graduate	1.0	[0.9, 1.2]		0.9	[0.8, 1.1]		1.3	[1.2, 1.5]	***	0.9	[0.7, 1.1]		1.0	[0.8, 1.3]	
Some college/associate’s degree	1.2	[1.0, 1.4]	**	0.9	[0.7, 1.0]		1.4	[1.2, 1.6]	***	0.8	[0.7, 0.9]	***	0.9	[0.8, 1.1]	
College graduate	1.4	[1.1, 1.6]	***	0.9	[0.7, 1.1]		1.2	[1.1, 1.5]	**	0.6	[0.5, 0.7]	***	0.7	[0.5, 0.9]	**
Marital Status(Married = reference)															
Widowed	0.8	[0.7, 1.0]		1.5	[1.3, 1.8]	***	1.2	[1.0, 1.4]	*	1.1	[0.9, 1.3]		1.3	[1.0, 1.6]	*
Divorced or separated	1.3	[1.2, 1.4]	***	1.2	[1.0, 1.4]	**	1.0	[0.9, 1.1]		1.0	[0.9, 1.1]		1.4	[1.1, 1.7]	**
Never been married	1.1	[1.0, 1.2]		1.0	[0.9, 1.2]		0.9	[0.8, 1.0]	*	0.9	[0.8, 1.0]	*	1.0	[0.8, 1.3]	
Poverty Level (Living in poverty = reference)															
Income up to twice the federal poverty level	1.2	[1.1, 1.3]	**	1.3	[1.1, 1.6]	**	1.0	[0.9, 1.1]		1.1	[1.0, 1.3]		1.5	[1.3, 1.9]	***
Income greater than twice the federal poverty level	1.0	[0.9, 1.1]		1.2	[1.0, 1.4]		1.1	[1.0, 1.2]		1.3	[1.2, 1.5]	***	1.3	[1.1, 1.5]	**
Population Density ^c^ (Not in CBSA = reference)															
CBSA > 1 million	1.1	[0.9, 1.3]		0.8	[0.7, 0.9]	**	0.9	[0.8, 1.1]		0.9	[0.8, 1.1]		0.9	[0.8, 1.1]	
CBSA < 1 million	1.1	[1.0, 1.3]		0.9	[0.7, 1.1]		1.0	[0.9, 1.1]		1.0	[0.9, 1.2]		0.9	[0.8, 1.1]	
HIV/AIDS ever (Never/unknown = reference)	0.8	[0.3, 2.1]		2.6	[0.7, 9.9]		1.8	[0.6, 5.4]		1.9	[0.7, 5.6]		1.1	[0.3, 5.0]	
Body Mass Index	1.0	[1.0, 1.04]	***	1.0	[1.0, 1.03]	***	1.1	[1.1, 1.1]	***	1.1	[1.1, 1.1]	***	1.0	[1.0, 1.04]	***
Past-year mental illness ^d^ (None = reference)															
Mild	1.3	[1.2, 1.5]	***	1.6	[1.4, 1.9]	***	1.4	[1.2, 1.5]	***	1.4	[1.2, 1.6]	***	1.9	[1.6, 2.3]	***
Moderate	1.7	[1.5, 2.0]	***	1.8	[1.5, 2.1]	***	1.4	[1.2, 1.6]	***	1.4	[1.2, 1.7]	***	2.3	[1.8, 2.9]	***
Serious	2.1	[1.9, 2.4]	***	2.1	[1.8, 2.5]	***	1.9	[1.6, 2.2]	***	1.7	[1.4, 2.1]	***	2.1	[2.6, 3.8]	***
Past-month nicotine dependence (Not dependent = reference)	0.9	[0.8, 1.1]		0.9	[0.8, 1.0]		1.0	[0.9, 1.1]		1.2	[1.0, 1.4]		2.8	[2.4, 3.1]	***
Past-year alcohol dependence/abuse (Not dependent/abusing = reference)	1.0	[0.8, 1.2]		0.9	[0.7, 1.1]		1.0	[0.9, 1.2]		0.7	[0.5, 1.0]	*	0.7	[0.6, 1.0]	*
Past-year other drug dependence/abuse (Not dependent/abusing = reference)	1.3	[1.0, 1.5]	*	1.6	[1.2, 2.1]	**	1.4	[1.1, 1.8]	*	1.0	[0.7, 1.4]		1.5	[1.1, 2.0]	**
Model Statistics and Diagnostics															
F_(31, 20)_^e^	50.4	***		59.9	***		145.5	***		75.6	***		50.9	***	
Squared Residuals ^f^	−4.20	NS		−0.80	NS		−6.20	NS		−1.45	NS		0.06	NS	
Coefficient of Discrimination ^g^	−47.0	***		−110.0	***		−160.0	***		−120.0	***		−90.1	***	
	**Kidney Disease**			**Cancer**			**Hepatitis B or C**			**Sexually Transmitted Infections ^a^**			**HIV/AIDS**		
Sexual Orientation (Heterosexual = reference)															
Bisexual	1.2	[0.6, 2.5]		1.9	[1.0, 3.3]	*	2.8	[1.1, 7.5]	*	2.1	[1.3, 3.6]	**	0.5	[0.1, 5.1]	
Gay	0.5	[0.1, 4.0]		1.5	[0.5, 4.6]		2.2	[0.3, 16.0]		1.9	[0.5, 7.1]		0.3	[0.004, 14.6]	
Sexual attraction ^b^	1.0	[0.7, 1.3]		0.9	[0.7, 1.1]		0.9	[0.6, 1.4]		0.8	[0.6, 1.1]		1.7	[0.7, 4.2]	
Discordant sexual identity and attraction (Condordant = reference)	1.3	[0.7, 2.4]		1.1	[0.7, 1.7]		1.1	[0.6, 2.3]		2.2	[1.5, 3.1]	***	0.7	[0.1, 6.4]	
Sexual Identity and Concordance Interaction (concordant + bisexual discordant + gay discordant = reference)															
Bisexual and Discordant	2.1	[0.4 11.7]		0.5	[0.1, 2.4]		1.7	[0.3, 9.5]		0.2	[0.1, 0.5]	**	1.0	NE	
Gay and Discordant	1.7	[0.2, 15.2]		0.8	[0.3, 1.9]		0.8	[0.2, 4.6]		0.2	[0.1, 0.8]	*	1.1	[0.01, 97.7]	
Race/ethnicity (White = reference)															
Non-Hispanic black/African-American	1.2	[0.8, 1.6]		0.4	[0.3, 0.5]	***	0.7	[0.5, 1.1]		1.5	[1.2, 1.8]	**	3.2	[1.3, 7.9]	*
Hispanic	1.1	[0.7, 1.4]		0.5	[0.4, 0.6]	***	1.1	[0.8, 1.6]		0.9	[0.7, 1.2]		1.3	[0.2, 6.6]	
Asian/Pacific Islander/Native American/multi-ethnic	0.9	[0.6, 1.4]		0.6	[0.4, 0.7]	***	1.6	[1.1, 2.3]	*	0.7	[0.6, 0.9]	*	1.8	[0.4, 8.2]	
Age in years (18–25 = reference)															
26–34	1.5	[1.1, 2.1]		2.7	[2.0, 3.7]	***	2.3	[1.3, 3.8]	**	0.8	[0.7, 1.0]	*	0.9	[0.3, 3.3]	
35–49	2.7	[1.9, 3.8]	**	6.1	[4.6, 8.0]	***	4.0	[2.3, 6.9]	***	0.6	[0.5, 0.8]	***	3.6	[1.2, 11.4]	*
50–64	4.3	[3.0, 6.2]	***	15.1	[11.1, 20.6]	***	7.9	[4.4, 14.3]	***	0.5	[0.4, 0.7]	***	5.6	[1.8, 17.6]	**
65+	11.1	[7.8, 15.8]	***	31.8	[23.4, 43.3]	***	8.9	[4.6, 17.5]	***	0.4	[0.3, 0.5]	***	1.3	[0.3, 5.2]	
Education level (Less than high school = reference)															
High school graduate	0.9	[0.7, 1.2]		1.3	[1.1, 1.7]	*	0.9	[0.6, 1.3]		1.3	[1.0, 1.7]	*	0.3	[0.1, 0.7]	**
Some college/associate’s degree	1.1	[0.8, 1.5]		1.6	[1.2, 2.0]	**	0.8	[0.6, 1.2]		1.2	[1.0, 1.5]		0.2	[0.1, 0.7]	*
College graduate	0.8	[0.6, 1.1]		1.7	[1.3, 2.2]	***	0.9	[0.6, 1.3]		1.4	[1.1, 1.9]	**	0.0	[0.0, 0.2]	***
Marital Status (Married = reference)															
Widowed	1.2	[0.9, 1.6]		1.1	[0.9, 1.2]		1.3	[0.9, 2.1]		0.8	[0.5, 1.3]		3.4	[1.0, 12.4]	*
Divorced or separated	1.1	[0.9, 1.5]		1.1	[1.0, 1.3]	*	1.1	[0.9, 1.5]		2.0	[1.6, 2.6]	***	3.9	[1.3, 11.6]	*
Never been married	1.0	[0.8, 1.4]		1.1	[0.9, 1.4]		1.2	[0.8, 1.8]		1.5	[1.3, 1.8]	***	3.4	[1.1, 10.5]	*
Poverty Level (Living in poverty = reference)															
Income up to twice the federal poverty level	1.5	[1.1, 2.0]	*	0.8	[0.7, 1.0]		2.3	[1.5, 3.4]	***	1.1	[0.9, 1.3]		1.3	[0.5, 3.1]	
Income greater than twice the federal poverty level	1.4	[1.2, 1.7]	**	1.0	[0.8, 1.1]		1.2	[0.8, 1.7]		1.1	[0.9, 1.3]		2.0	[0.9, 4.6]	
Population Density ^c^ (Not in CBSA = reference)															
CBSA > 1 million	0.8	[0.6, 1.3]		1.2	[1.0, 1.4]	*	1.3	[0.8, 2.1]		1.0	[0.8, 1.4]		3.9	[1.1, 13.9]	*
CBSA < 1 million	0.9	[0.7, 1.3]		1.2	[1.0, 1.4]	*	1.4	[0.9, 2.3]		1.1	[0.8, 1.4]		2.1	[0.6, 8.0]	
HIV/AIDS ever (Never/unknown = reference)	6.0	[1.0, 35.1]		1.7	[0.4, 7.5]		10	[2.6, 38.1]	***	4.2	[1.5, 12.0]	**	NE		
Body Mass Index	1.0	[1.0, 1.04]	*	1.00	[1.0, 1.0]		1.0	[1.0, 1.0]	*	1.0	[1.0, 1.0]		1.0	[0.9, 1.0]	
Past-year mental illness ^d^ (None = reference)															
Mild	1.9	[1.4, 2.4]		1.2	[1.0, 1.4]		2.0	[1.5, 2.7]	***	1.7	[1.4, 2.1]	***	2.4	[0.7, 8.8]	
Moderate	2.0	[1.4, 3.0]		1.3	[1.0, 1.6]	*	1.9	[1.2, 2.9]	**	1.6	[1.3, 2.9]	***	4.5	[1.9, 10.9]	***
Serious	2.9	[2.0, 4.2]	***	1.8	[1.5, 2.2]	***	1.8	[1.1, 2.7]	*	1.9	[1.6, 2.3]	***	1.4	[0.5, 4.3]	
Past-month nicotine dependence (Not dependent = reference)	0.8	[0.6, 1.0]		1.0	[0.8, 1.1]		1.6	[1.2, 2.2]	***	1.3	[1.1, 1.5]	*	1.2	[0.4, 4.2]	
Past-year alcohol dependence/abuse (Not dependent/abusing = reference)	0.3	[0.2, 0.6]	**	0.9	[0.7, 1.3]		1.3	[0.8, 2.0]		1.8	[1.5, 2.2]	***	2.2	[0.9, 5.3]	
Past-year other drug dependence/abuse (Not dependent/abusing = reference)	1.0	[0.6, 1.8]		1.1	[0.8, 1.6]		4.0	[2.6, 6.2]	***	1.8	[1.5, 2.1]	***	1.2	[0.3, 4.5]	
Model Statistics and Diagnostics															
F_(29, 22)_^e^	27.3	***		34.9	***		18.2			14.2	***		47.2	***	
Squared Residuals ^f^	1.00	NS		0.25	NS		1.07	NS		−1.83	NS		0.60	NS	
Coefficient of Discrimination ^g^	−44.9	***		−96.1	***		−50.0	***		−46.7	***		−24.4	***	

Note. Bivariate logistic regression models are based on analysis NSDUH 2015–2017 data obtained from 67, 648 female respondents 18 years of age and older. Owing to missing data on covariates, the unweighted N for HIV/AIDS was 63, 346 and 63, 495 for all other medical conditions. Data were weighted to adjust for variation in sampling probabilities. Standard error estimates and significance levels account for design effects owing to stratification and clustering. ^a^ Sexually transmitted infections includes gonorrhea, chlamydia, syphilis, or herpes. ^b^ Sexual attraction was self-reported using a scale from 1 to 5 with the lowest score indicating exclusive attraction to members of the opposite sex and higher scores indicating increasingly greater attraction to the same sex. ^c^ Population density is based on Core Based Statistical Areas (CBSA), which are used by the U.S. Office of Management and Budget to define population centers in the U.S. ^d^ Past-year mental illness category is based thresholds as derived from a logistic regression model of mental illness as predicted by age, functional disability, thoughts of suicide, and major depressive episode. ^e^ Reflects an F statistic testing improvement in model fit with included parameters versus a model with only the constant term. Significance indicates a statistically reliable improvement in model fit with inclusion of the parameter estimates. ^f^ Reflects a t-test of the squared residuals term after fitting the main effects model. Significance indicates unexplained residual variance for the main effects only model and potential model misspecification. ^g^ Based on the mean difference between the predicted probabilities of having a condition for observed cases and non-cases. Significance was assessed using a t-test. NS = Not significant; NE = Not estimated; * *p* < 0.05, ** *p* < 0.01; *** *p* < 0.001.
